# Coastal engineering infrastructure impacts Blue Carbon habitats distribution and ecosystem functions

**DOI:** 10.1038/s41598-022-23216-7

**Published:** 2022-11-11

**Authors:** Inés Mazarrasa, Jordi Garcia-Orellana, Araceli Puente, José A. Juanes

**Affiliations:** 1 IHCantabria - Instituto de Hidráulica Ambiental de La Universidad de Cantabria, Universidad de Cantabria, Parque Científico y Tecnológico de Cantabria (PCTCAN), C/ Isabel Torres 10, Santander, Spain; 2 Centro de Estudios Avanzados de Blanes, Consejo Superior de Investigaciones Científicas, Blanes, Girona, Spain; 3 Departament de Fısica, Universitat Autònoma de Barcelona, Bellaterra, Barcelona, Spain; 4 Institut de Ciència I Tecnologia Ambientals, Universitat Autònoma de Barcelona, Bellaterra, Barcelona, Spain

**Keywords:** Biogeochemistry, Environmental sciences

## Abstract

Intertidal estuarine habitats (e.g., saltmarshes and tidal flats) provide important ecosystem services to society, including coastal protection, food provision and C_org_ sequestration. Yet, estuaries and estuarine habitats have been subjected to intense human pressure, such as land-use change and artificialization of the shoreline to support economic activities and uses. Construction of engineering infrastructures (e.g., piers, bridges) in these areas alters estuary-wide hydromorphological conditions and thus sedimentation patterns at the estuarine scale, which are key drivers of habitats distribution and ecosystem structure, processes and functions. Most of the research on the impact of civil engineering structures on coastal habitats has focused on the biological communities that colonize them or the bottoms where they are placed, whereas their indirect impacts on adjacent habitats has been largely unexplored. Understanding the influence of man-made infrastructures on the distribution of estuarine habitats and functions is critical, particularly considering that shoreline armoring is expected to increase as a way to protect coastal areas from hazards derived from climate change. Shifts in habitat distribution and functions occur in several years or decades and relating them with the occurrence of past historical events is challenging when no monitoring data is available. By examining historical aerial photographs and different biogeochemical properties along a saltmarsh soil record, this study demonstrates that the construction of an infrastructure (i.e. bridge) caused a rapid transformation (~ 30 years) of a bare sandflat into a high marsh community and to significant changes in sediment biogeochemical properties, including the decrease in sediment accretion rate and C_org_ burial rates since then. This study contributes to increase the knowledge on the impact that the construction in coastal areas of civil engineering infrastructures can cause in intertidal habitats distribution and the ecological functions they provide for climate change adaption and mitigation.

## Introduction

Coastal areas have historically supported the development of human civilizations around the world, concentrating a high density of population and human activities^1^. More than 60% of the world’s population lives in the coastal zone despite the fact that it occupies less than 15% of the Earth’s land surface^2^. In particular, estuaries have been focal points of human settlement and commerce and host many large coastal cities worldwide^3^.

Estuaries contain important habitats that provide multiple ecosystem services to society, including food provision, biodiversity maintenance and cultural and recreational values^4^. Estuarine vegetated habitats, such as saltmarshes, have received special attention for their role in climate change mitigation and adaptation^5^. They are significant organic carbon (C_org_) sinks, being able to bury organic carbon (C_org_) at rates comparable to terrestrial forests^6^, by accumulating large living and dead biomass in the soil compartment (known as autochthonous C_org_) and trapping organic particles from the water column derived from adjacent ecosystems (i.e., allochthonous C_org_)^7^. Their high C_org_ sequestration and storage capacity place them within the so-called group of Blue Carbon ecosystems, along with mangroves and seagrass meadows^8^. In addition, saltmarsh canopy buffers wave energy and currents, enhancing particle sedimentation from the water column and avoiding erosion, protecting coastal areas from extreme weather events and stabilizing the shoreline^9^. As a result of the sedimentation of inorganic and organic particles from the water column together with the increase of belowground plant biomass and detritus, saltmarsh soils tend to accrete vertically^10^. Soil accretion confers this ecosystem the capacity to adapt to sea-level rise^11^ and to maintain all the ecosystem services they provide under future climate change scenarios^12^. At a lower intertidal range compared to saltmarshes, bare tidal (sand or mud) flats also provide important ecosystem services to coastal communities, such as the support of fisheries^13^ and biodiversity, being essential habitats for the survival of resident and migratory birds that feed on invertebrates that inhabit these intertidal habitats^14,15^. Due to their lack of vegetation, soil C_org_ stocks by surface area in bare tidal flats are usually lower than in adjacent saltmarsh communities^16^. Yet, due to their lower position relative to tidal range, tidal flats experience more frequent and longer hydroperiods and thus higher sediment accumulation than saltmarsh communities^17^. As a consequence, bare tidal flats may show sediment accretion rates and allochthonous C_org_ burial rates comparable to those shown by saltmarshes and other estuarine macrophytes such as seagrass meadows^16,18^. In addition, and due to the large surface area bare tidal flats occupy within estuaries, they can store significant C_org_ stocks at the estuarine scale^16^.

Despite all the benefits that estuarine habitats provide to societies, they are among the most threatened on the planet, mainly due to high pressure of human activities in coastal areas^19^. To meet with social demands and facilitate economic activities, estuaries have been intensively modified by humans through reclamation and transformation of tidal areas to other uses and the construction of artificial infrastructures such as pier pilings, breakwaters, outfall pipelines and bridge supports^20^. Shoreline armoring has been accelerated in recent decades due to the need to protect coastal areas against threats related to climate change, such as the increased frequency and intensity of extreme weather events or sea level rise^21,22^. Shoreline armoring is considered one of the most important threats to the natural functioning of estuaries and estuarine habitats, along with pollution, or land reclamation^23^.

The construction of civil engineering infrastructures in coastal areas significantly alters the natural environment^23^. Most of them are constructed on soft sediments, including dunes and mudflats, causing at first, the loss of the habitat surface on which the infrastructure is constructed and changes on the biological zonation patterns^22^. The construction of civil engineering infrastructures can also affect habitats located far from the infrastructure^22^ as they usually modify the hydrodynamic regime of the entire system, including wave energy, tidal prism, currents and riverine flow^22^, causing changes in hydroperiod and sedimentary process^24^, which are key factors in the development and distribution of intertidal estuarine habitats^17,25^. For instance, enhanced depositional conditions and increased sedimentation of fine particles can trigger the transformation of intertidal mudflats into saltmarshes, when other conditions for the establishment and germination of pioneer saltmarsh plant species are met^26^. On the contrary, increasing hydroperiods and water currents due to modification of river channels can cause very rapid and extensive erosion and loss of saltmarshes and their conversion to unvegetated mudflats^27^. Shifting from one type of habitat to other leads to changes in ecosystem processes and functions^28^. On the other hand, changes in hydrological regimes induced by coastal infrastructures can also induce changes in C_org_ sequestration rates and sediment accretion in a particular habitat by altering the input of mineral and organic particles. For instance, the presence of dykes or stonewalls has been shown to lead to lower sediment accretion rates and soil C_org_ stocks in embanked saltmarshes compared to natural saltmarshes, due to the partial restriction of tidal flow^29^.

Despite the widespread construction of civil engineering infrastructures in estuarine areas, very little research has been conducted on the impacts on the aquatic habitats, particularly when compared to the research effort invested in assessing the effects of urbanization on terrestrial ecosystems^22,30^. Most of research on the impact of civil engineering structures on coastal habitats has been focused on the biological communities that colonize them or the sediment where they are placed^20^, whereas their impact on adjacent habitats, has been largely unexplored. Shoreline armoring in estuarine areas is predicted to increase as a way to protect coastal areas against the impacts of climate change. At the same time, the restoration and conservation of estuarine habitats is increasingly been recognized as a key element in the development of sustainable and efficient climate change adaptation and mitigation strategies, thanks to the role they play in coastal protection and C_org_ sequestration^31,32^. Within this context, understanding the influence of artificial infrastructures construction on the distribution and functions of estuarine habitats is critical to identify the best and most sustainable design^23^. Shifts in habitat distribution and functions may take from years to decades. Thus, relating these changes with events that occurred in the past is a challenge when no monitoring in the areas has been conducted. The analysis of coastal ecosystems dated soil cores has been demonstrated to be a very good tool to identify changes in environmental conditions and habitats along time and relate them to historical events^33^.

**Figure 1:**
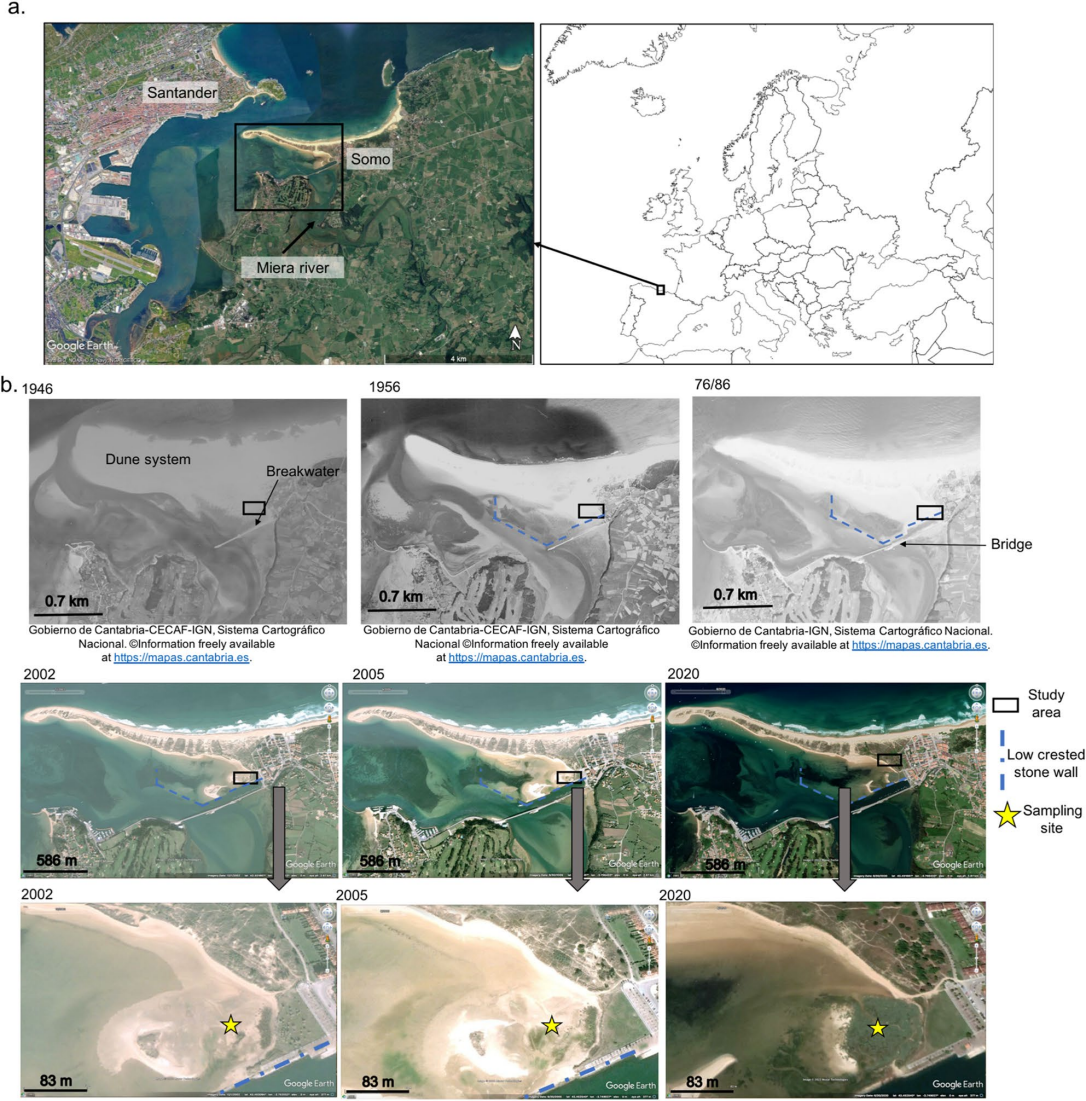
Study site: (**a**) Location of the study area in the Bay of Santander estuary, in the north of the Iberian Peninsula and (**b**) past sequential aerial photographs of the study area showing the location of the coastal infrastructures placed during the study period and the sampling site (43.452136°/ -3.748134°). Europe map was obtained from WIKIMEDIA COMMONS under the Creative Commons Attribution-Share Alike 3.0 Unported license. Aerial photographs were obtained from www.mapascantabria.com (years 1946, 1956, 1976/86) and Google Earth (years 2002, 2005 and 2019).

This study analyses and discusses the impact of the construction of a civil engineering infrastructure on the distribution and functions of adjacent estuarine habitats, focusing particularly on soil C_org_ sequestration and vertical accretion, key processes in the role these habitats play for climate change adaptation and mitigation. This study is based on the examination of old aerial photographs and biogeochemical properties along the depth profile of three replicate soil cores (encompassing the last ~110 years) sampled in a saltmarsh located at the mouth of an estuary that flows into the Bay of Santander (Gulf of Biscay) (Fig. 1a), where the building of a bridge in 1978 caused remarkable changes in sedimentation and hydrodynamics across both sides of the estuary^34^.

## Results

### Old aerial photographs

**Figure 2:**
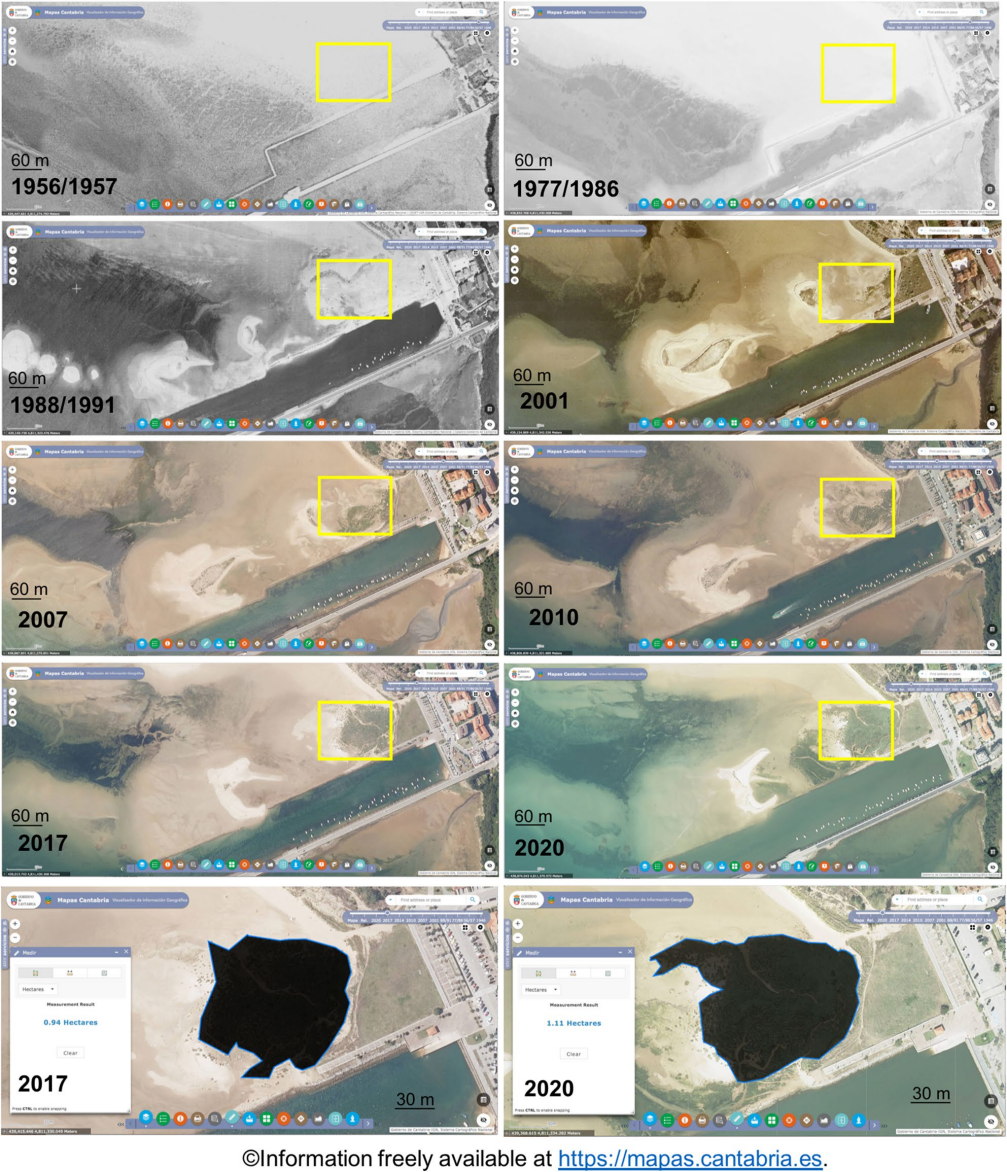
Old aerial photographs of the sampling area extracted from https://mapas.cantabria.es. No old aerial photograph is available for the year of sampling. The area occupied by the high marsh community at the time of sampling (year 2019) is estimated by measuring, through the measuring tool available in https://mapas.cantabria.es, the surface area occupied in 2017 and 2020.

Visual inspection of old aerial photographs shows that between 1946 (no previous aerial pictures of the area are available) and 1986 the study area was a tidal flat (Fig. 1b). At that time, a breakwater facilitated the loading of boats that transport goods to the main city (Santander). Photographs show that between 1946 and 1959 a low crested stone wall was built in front of the breakwater to facilitate navigation and presumably for the transformation of the tidal area within the wall to other uses. Yet, the area was not transformed and the low crested stone has remained to the present day. The old aerial photographs (Figs. 1b, 2) suggest a shift in the habitat of the study area after the building of the bridge in 1978, from a bare habitat to a vegetated one in approximately 0.9–1.1 ha (Fig. 2). At the time of this study in 2019, the sampling area was a high marsh community formed by the species *Halimione portulacoides*, which dominates mid to high marsh zones over extensive areas of European saltmarshes.

### Sediment properties

**Figure 3:**
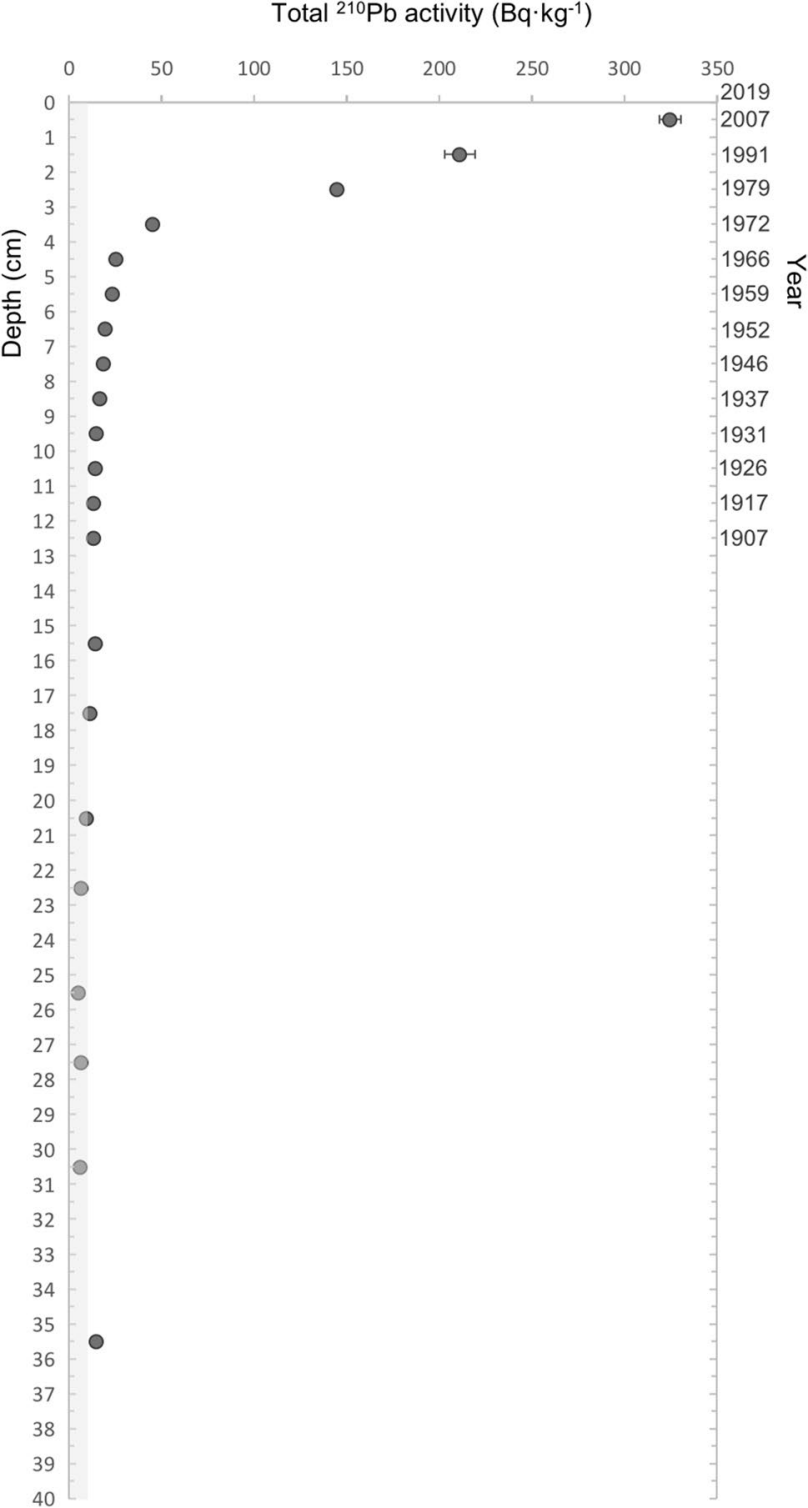
^210^Pb profile along sediment core: Excess ^210^Pb concentration (± standard error) along the depth profile (left y axis) and time (right y axis) of the core BS2A1. The grey area represents the supported ^210^Pb (9 ± 3 Bq kg^−1^).

The analysis of ^210^Pb_xs_ concentration along the sediment depth profile of one of the saltmarsh cores (BS2A1) sampled showed two clearly different exponential trends between the 0.0–3.5 cm and 3.5–13.0 cm sections, that indicates different sedimentation regimes (Fig. 3). The dating model applied (CF:CS) in both sections indicates that the change in the sedimentation pattern observed between centimeters 2.5 (1979 + -4) and 3.5 (1972 + -1) is consistent to the construction of the bridge in 1978. According to this model, the top 13 cm depth encompassed 112 years since core was sampled, in 2019 (Fig. 3).

Average (± se) sediment dry bulk density and C_org_ concentration along the sediment depth profiles of the three cores sampled ranged from 1.1 ± 0.1 g cm^−3^ to 1.3 ± 0.1 g cm^−3^ and from 1.3 ± 0.6%DW to 1.9 ± 0.4%DW, respectively (Table 1). Sediment mean grain size and the content of silt and clay were 277 ± 76 µm and 26 ± 8%DW, respectively and the average C_org_ isotopic signature (δ^13^C_org_) was − 24.4 ± 0.3 ‰ (Table 1). Potential sources of C_org_ to sediment in the sampling area, the saltmarsh species dominating the area (*Halimione portulacoides*) and seagrass (*Zostera noltei*) from a seagrass meadow nearby, showed C_org_ isotopic signatures (δ^13^C_org_) of − 27.3 ‰ and − 10.5 ‰, respectively (Table 2).

**Table 1:** Biogeochemical properties examined along the sediment depth and across accumulation periods in the three saltmarsh sediment cores analyzed (BS2A1, BSA2 and BSA3).

CORE	Variable	All core	After bridge (1978–2019; 0–3 cm depth)		Before bridge (1907–1978; > 3 cm depth)	
			Mean ± se (n)	Min–Max	Mean ± se (n)	Min–Max
BS2A1	DBD (g cm^−3^)	1.32 ± 0.07 (31)	0.39 ± 0.03 (3)	0.36–0.46	1.42 ± 0.05 (28)	0.87–1.75
	TOC (%DW)	1.94 ± 0.4 (21)	9.4 ± 1.5 (3)	6.7–11.8	0.71 ± 0.13 (18)	0.1–1.9
	Mean grain size (µm)	243 ± 44 (10)	16.5 (2)	15.1–17.9	299 ± 29 (8)	127–372
	Silt & Clay (%)	31 ± 11 (10)	94 (2)	93–95	15 ± 5 (8)	1.6–46.7
	Sand (%)	69 ± 11 (10)	6.2 (2)	5.3–7.1	85 ± 5 (8)	53–98
	SAR (cm y^−1^)	0.13 ± 0.01 (13)	0.08 ± 0.02 (4)	0.04–0.14	0.15 ± 0.01 (9)	0.10–0.20
	C_org_ burial (mg cm^−2^ y^−1^)	1.7 ± 0.21 (13)	2.5 ± 0.3 (4)	1.7–3.0	1.3 ± 0.2 (9)	0.4–2.0
	δ^13^C (%)	− 24.3 ± 0.3 (17)	− 26.34 ± 0.03 (3)	− 26.4–(− 26.3)	− 23.8 ± 0.2 (14)	− 24.9(–)− 22.9
BS2A2	DBD (g cm^−3^)	1.1 ± 0.1 (12)	0.6 (2)	0.58–0.62	1.1 ± 0.1 (11)	0.58–1.53
	TOC (%DW)	1.4 ± 0.4 (11)	3.5 (2)	2.4–4.5	1.1 ± 0.2 (10)	0.4–2.4
BS2A3	DBD (g cm^−3^)	1.3 ± 0.1 (13)	0.9 (2)	0.52–1.18	1.4 ± 0.08 (12)	0.94–1.93
	TOC (%DW)	1.3 ± 0.6 (11)	4.5 (2)	1.4–7.5	0.7 ± 0.1 (10)	0.1–1.4

**Table 2:** C_org_ isotopic signature (δ^13^C_org_) and C:N ratio of potential sources to soil C_org_ stocks measured in this study. N measurements indicated in brackets.

Source	δ^13^C_org_
Saltmarsh (*Halimione portulacoides*)	− 27.3 (2)
Seagrass (*Zostera noltii*)	− 10.5 (2)

**Figure 4:**
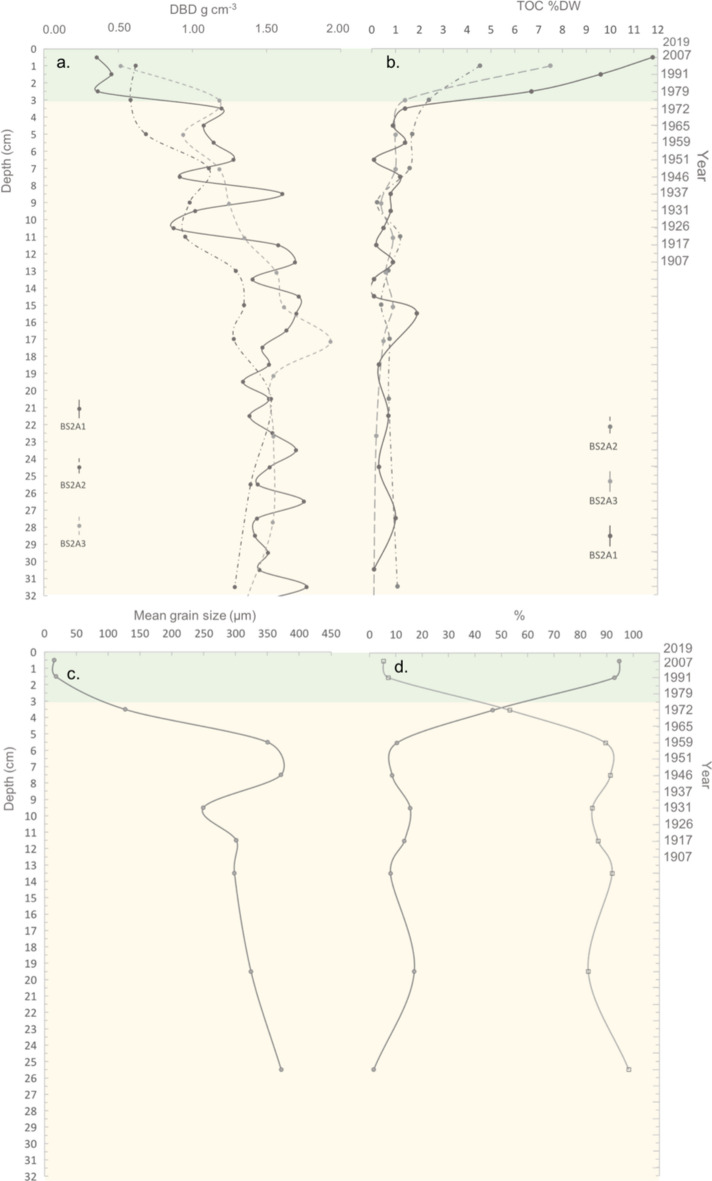
Changes in biogeochemical variables along sediment depth (left axis) and time (right axis): (**a**) dry bulk density and (**b**) sediment organic carbon content measured in the three sediment cores sampled (BS2A1, BS2A2 and BS2A3) and (**c**) mean grain size and (**d**) proportion of sand (•—) and silt & clay (•—) measured in the core BS2A1. Green color indicates the period after the building of the bridge in 1978.

**Figure 5:**
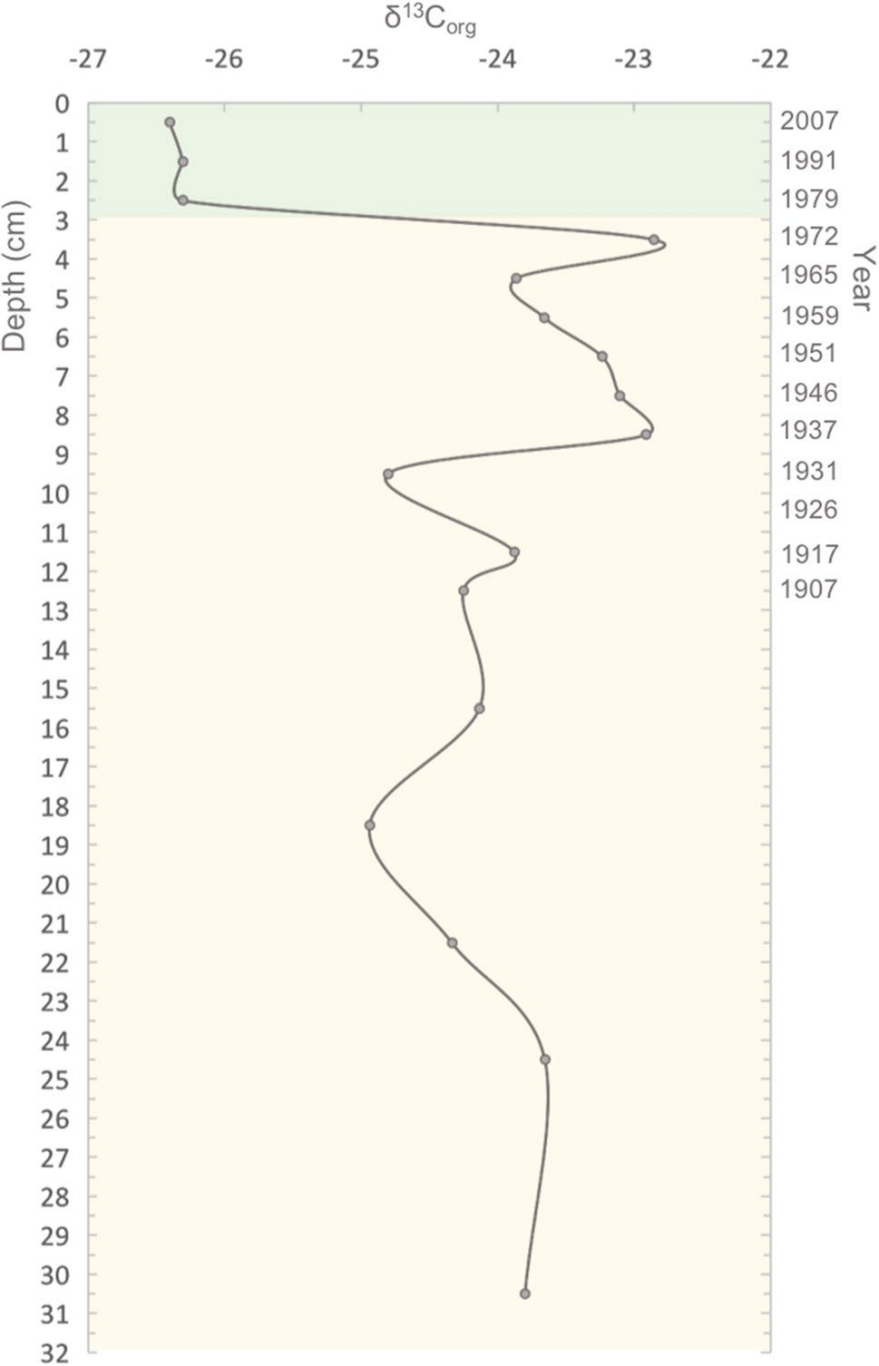
Changes in δ^13^C_org_ along sediment depth (left axis) and time (right axis) in the core BS2A1**.** Green color indicates the period after the building of the bridge in 1978.

**Figure 6:**
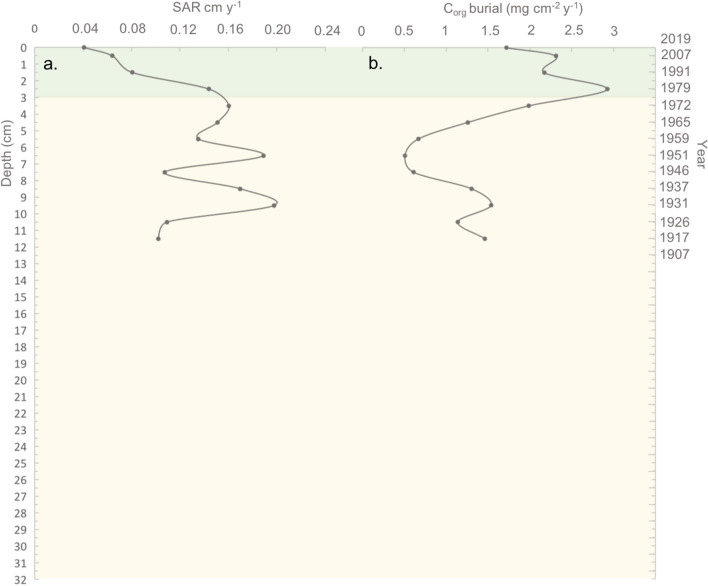
Changes in biogeochemical variables along sediment depth (left axis) and time (right axis) in the core BS2A1: (**a**) sediment accretion rate and (**b**) organic carbon burial rate. Green color indicates the period after the building of the bridge in 1978.

All biogeochemical variables changed along the sediment depth profile of the cores analyzed and differed across the two periods examined: before and after the building of the bridge in 1978 (Table 1, Figs. 4, 5 and 6). An abrupt shift in dry bulk density and C_org_ content was observed in the three cores examined between the 2.5–3.5 cm sediment depth sections accumulated in 1979 and 1972, respectively. Average sediment dry bulk density of each core was between two to three times lower (0.4 – 0.9 g cm^−3^) in the upper 3 cm, which corresponds to those sediments accumulated since the bridge construction in 1978, compared to that found in deeper sediments accumulated before (1.1 – 1.4 g cm^−3^) (Table 1, Fig. 4a). Average sediment C_org_ content (%DW) of each core was more than three times higher in the upper 3 cm (3.5–9.4%DW), accumulated since the bridge was built in 1978, compared to deeper sediments accumulated earlier (0.7–1.1% DW) (Table 1, Fig. 4b).

Sediment mean grain size was one order of magnitude lower in the upper 3 cm (16.5 µm) accumulated after bridge construction compared to the deeper sediments accumulated before (300 ± 30 µm). Consistently, the content of mud (i.e. silt & clay) and sand were higher and lower (93.8% and 6.2%), respectively, in the upper 3 cm, compared to deeper sediments (15.1% and 84.9%, respectively) (Table 1, Fig. 4c,d). C_org_ isotopic signature (δ^13^C_org_) showed an abrupt drop between the 3.5–2.5 cm depth sections accumulated in 1972 and 1979, respectively (Fig. 5). The upper 3 cm sediments, accumulated since the building of the bridge, showed a lower δ^13^C_org_ (− 26.33 ± 0.03 ‰) than deeper sediments (− 23.81 ± 0.17 ‰) accumulated before the building of the bridge (Table 1).

Sediment accretion rate (SAR) of sediments accumulated before the building of the bride in 1978 (3–13 cm depth) was one order of magnitude higher (0.14 ± 0.02 cm·y^−1^, avg. ± se) than that found in the sediments accumulated after the building of the bridge (top 3 cm depth) (0.04 ± 0.01 cm·y^−1^, avg. ± se) (Table 1, Fig. 6a). In addition, before the building of the bridge, SAR ranged from 0.1 cm·y^−1^ to 0.2 cm y^−1^without showing a clear trend whereas a clear decreasing trend is shown for the sediment accumulated since the bridge was built towards present (Fig. 6a). The average C_org_ burial rate estimated since the bridge construction (top 3 cm depth) (2.3 ± 0.5 mg C_org_ cm^−2^·y^–1^), doubled that found for the previous period (1.2 ± 0.1 mg C_org_ cm^−2^·y^−1^) (Table 1, Fig. 6b). However, C_org_ burial rate showed different trends along the sediment depth profile and across periods, tending to decrease from 12 cm depth towards 7 cm depth (comprising the period 1917—1951), shifted to an increasing trend between 7 cm depth and 3 cm depth (comprising the period between 1951 and 1978) and shifted to a decreasing trend again from 3 cm towards sediment surface (i.e., from 1978 towards present).

## Discussion

The sediment cores examined showed significant changes along the sediment depth profile in all biogeochemical variables examined, particularly when comparing sediments accumulated before and after the bridge construction. The trends observed are consistent with the shift in habitat at the study site from an unvegetated tidal flat to a saltmarsh community, suggested in the old aerial photographs.

Sediments accumulated before the bridge construction in 1978 (> 3 cm) were representative of those of an unvegetated tidal sandflat, with high DBD (1.1 – 1.4 g cm^−3^) and low C_org_ content (0.7–1.1 C_org_ %DW)^18,35^. Sediment grain size was mainly dominated by sand (85%), typical of outer estuarine sections and high energy environments^36,37^.

After the building of the bridge in 1978, the biogeochemical properties of sediments changed significantly. Sediment C_org_ content increased to values of 3.5–9.4 C_org_ %DW and DBD decreased to 0.4–0.9 g cm^−3^ reaching values comparable to those found in saltmarshes elsewhere (5.4–8.7 C_org_ %DW; 0.24–0.9 mg cm^−3^, respectively^38,39^). Sediment shifted from sandy (85% of sands) to muddy (94% of silt and clay). A high content of silt and clay is indicative of coastal environments with low hydrodynamic energy compared to areas with a low content of silt and clay^40^. The dramatic shift towards a mud dominated sediment observed in the study area reflects a reduction in the hydrodynamic energy and the transformation of the site into a more depositional environment^41^. The increase in the content of silt and clay seems to have started around 1959, before the building of the bridge (Fig. 3d), although it was not until the building of the bridge in 1978 that sediment become mainly dominated by mud. The earlier increase in the content of silt and clay could be due to the building of the low crested stone wall sometime between 1946 and 1959, that can be observed in the aerial photographs (Fig. 1b). Thus, it is likely that the accumulation of fine sediment, favored by the creation of a more depositional environment due to the construction of the stone wall and then the bridge, favored the settlement and germination of saltmarsh vegetation and the evolution of the tidal sandflat into a saltmarsh^26^. This hypothesis is supported by the drop in the soil C_org_ isotopic signature observed after the building of the bridge (from − 23.3 ‰ to − 26.3 ‰) towards that measured in the saltmarsh species present in the area at the time of sampling (*Halimione portulacoides*; δ^13^C_org_ = − 27.3 ‰) which suggests an increase in the contribution of saltmarsh derived C_org_ to soil stocks since the bridge construction. Despite C_org_ isotopic signature of saltmarsh biomass was measured in a limited number of samples in this study (n = 2), the values obtained are consistent to those reported for this species in the literature across different European estuaries (δ^13^C_org_ = − 25.3% to − 28.6%;^42,43^). Other potential C_org_ sources to soil stocks in the study area are phytoplankton, intertidal seagrass (*Zostera noltii and Zostera marina)* meadows, and organic matter from terrestrial macrophytes transported by the river. δ^13^C_org_ of marine phytoplankton (− 20.3 (-)− 22.4 ‰) and intertidal seagrasses (− 10.5 ‰ (-)− 14.5 ‰; SI Table 1) measured by this and previous studies^44,45^ are higher than that found in sediments accumulated before and after the building of the bridge suggesting that these sources of C_org_ could not have contributed to the significant drop in the δ^13^C_org_ observed in the most recent period. δ^13^C_org_ of organic matter from terrestrial plants (− 24.8 (-)− 27.9 ‰)^44^ is similar to that of the saltmarsh species in the area of study and to that found in the soil accumulated after the building of the bridge. Yet, considering the increase in vegetation cover shown by the old aerial photographs in the sampling area, the most plausible explanation to the drop in the soil δ^13^C_org_ observed after the building of the bridge, is the accumulation of saltmarsh plant biomass and detritus.

The change in hydrodynamic conditions and subsequent habitat shift lead to changes in the sediment accretion and C_org_ burial rates of the site examined across periods. SAR dropped from an average of 0.14 ± 0.02 cm y^−1^ to 0.04 ± 0.01 cm y^−1^ after the bridge construction and shows a negative trend since then. C_org_ burial rate was higher, on average, after the building of the bridge (2.3 ± 0.3 mg cm^−2^ y^−1^) compared to the previous period (1.2 ± 0.2 mg cm^−2^ y^−1^), reflecting the significantly larger C_org_ stocks derived from belowground biomass accumulated by the saltmarsh vegetation, compared to bare tidal flats^16,46^. However, C_org_ burial rate tends to decrease towards present since the building of the bridge. The decreasing trends in SAR and C_org_ burial rate found in the sediments accumulated after the bridge construction reflect a decrease in the input of organic and mineral particles from the water column, a key factor for soil vertical accretion and C_org_ sequestration in saltmarshes^47,48^. The decrease in sediment input from the water column is consistent with the construction of the bridge, which is known to have acted as a barrier to natural tidal and river flow, leading to restrictions in flow dynamics and sedimentation between the inner and the outer section of the estuary^34^. Rivers and streams are important sources of organic and mineral particles to estuarine ecosystems^49^. The river influence determines temporal and spatial variability in C_org_ burial and sediment accretion rates across estuarine habitats (e.g. along marsh zonation) and locations (e.g. inner vs. outer estuarine sections)^49^. The results found in this study are consistent with previous studies reporting a decrease in estuarine habitats C_org_ burial and sediment accretion rates caused by artificial barriers to water flow^29,50^. In addition, the evolution of the tidal sandflat into a saltmarsh community after the bridge construction implied an increase in the relative position with regard to the tidal range and, as a consequence, a reduction of the frequency and duration of the inundation period^17^, which also contribute to explain the decrease in sediment accretion and C_org_ burial found. The lower hydroperiod in high marsh zones compared to adjacent tidal flats leads to a lower hydrodynamic energy in high marsh zones^46^, which is also consistent with the increase in the accumulation of fine particles observed towards present.

Sediment accretion rates is a key factor determining the capacity of saltmarshes to adapt to sea level rise^51^. When SAR does not match sea level rise, submergence can lead to large shifts in vegetation or even vegetation die-off^52^ causing significant changes or loss in ecosystem services provided, including C_org_ sequestration^28^. Sea level in Santander Bay is rising at a rate of 0.21 cm y^−1^ (years 1943–2004) according to recent estimates^53^, higher than the sediment accretion rate found in the study site, particularly after the building of the bridge (0.04 cm y^−1^). Thus, despite the building of the bridge leaded to the development of a saltmarsh and to an increase in the C_org_ stock and burial rate thanks to the accumulation of vegetation biomass, the bridge acts as a barrier to river flow and sediment input, causing this saltmarsh to be unable to match with predicted sea level and to be lost in the future^54^.

This study demonstrates that the building of the bridge triggered the transformation of an adjacent tidal sand flat into a high marsh community and led to an increase in the magnitude of the soil C_org_ stocks but to a decrease in SAR and C_org_ burial rates, determinant processes for the role saltmarshes play as C_org_ sinks in the long-term. We acknowledge that the conclusions of this study are based on a chronological model derived from a single dated core. Despite potential limitations derived from the lack of replicate dated cores, this is a common approach in paleoecological studies in coastal areas^33^ due to the difficulties and high costs associated to sediment dating in vegetated coastal habitats^55^. In this study, the similar trends observed in biogeochemical properties (i.e. DBD and TOC %DW) with depth in the two replicate non-dated cores along with the habitat shift observed in the old aerial photographs, support the conclusions derived from the dated core.

The results of this study highlight the need of considering the impact of the building of coastal engineering infrastructures in hydrodynamic processes and sedimentary patterns at the whole estuarine scale for planning ecosystem conservation and restoration projects that can be sustainable in the future. This is particularly important considering that shoreline armoring in estuarine areas is expected to increase as a way to protect coastal areas from impacts of climate change^23^ and that saltmarshes and other coastal vegetated habitats play a key role for climate change adaptation and mitigation through coastal protection and carbon sequestration.

## Materials and methods

### Study area

This study was conducted in the Bay of Santander, Gulf of Biscay (Fig. 1a). The Bay of Santander is one of the most important and largest estuaries along the northern coast of Spain, with a total extent of 22.5 km^2^^56^. As most estuaries in Europe, the Bay of Santander has experienced significant hydromorphological modifications^57^. The building of coastal infrastructures and the artificialization of the coastline have increased during the last century in order to support economic activities and connect the main city of Santander with other villages located along the Bay shoreline^57^. As a consequence, about 83% of the natural coastline of the estuary has disappeared, almost two-thirds of its intertidal area has been covered and over 40% of its volume has been lost^58^.

The study area is located at the mouth of the Miera estuary, next to the port of Somo, a touristic village located at the beginning of a sand-dune system that extents from east to west over approximately 5.5 km^59^ (Fig. 1b). At the time of this study in 2019, the sampling area was dominated by the saltmarsh species *Halimione portulacoides*, which dominates mid to high marsh zones over extensive areas of European saltmarshes.

### Soil core sampling and processing

In June 2019, three soil cores (30–32 cm long * 7 cm Ø) were extracted within an area of 25 m^2^ in the high marsh community of the study area (43.452136°/− 3.748134°; Fig. 1b) by manually hammering a PVC tube (60 cm L * 7 cm Ø). A negligible compression during sampling (0.8%) was detected in one of the cores, whereas no compression was detected in the other two cores. The cores were preserved frozen until processing.

One of the cores (BSA1) was sliced every 1 cm, whereas the other two cores (BS2A2, BS2A3) were sliced every 2 cm for the top 20 cm and every 5 cm for the deeper layers. Each sediment slice was measured for wet volume and dried at 60 ºC for a minimum of 72 h. The dry weight of each slice was measured and used together with wet volume to estimate sediment dry bulk density (DBD in g·cm^−3^).

### Biogeochemical analysis

Soil organic carbon content (C_org %_ DW) was measured in every depth section for the top 16 cm and in every other two depth sections for deeper sediments for the core sliced every 1 cm (BSA1) and in every depth section for the top 25 cm and in every other section for deeper sediments for the cores sliced at a lower resolution (BS2A2, BS2A3). C_org_ was analyzed in the IHLab Bio laboratory of the IHCantabria using a TC analyzer (Shimadzu TOC-L + SSM-5000A).

The following analysis (i.e. grain size, isotopic signature, radioactive dating) were performed only in the longest core sliced at a higher resolution (every 1 cm) (BS2A2).

Grain size analysis was performed in every depth section for the top 2 cm and in every other depth section along the sediment core at the Universitat de Barcelona with a Beckman Coulter LS GB500. The sediment was classified according to the Udden-Wentworth grain size scale (Ø: < 4 µm, clay; Ø: 4–63 µm, silt; Ø: 63–2000 µm, sand and Ø: 2000–4000 µm, gravel).

Organic carbon isotopic signature (δ^13^C_org_) (in pre-acidified subsamples) was measured in every depth section for the first 11 cm and in every other two sections for the rest of the sediment core using an Elemental Analyzer Flash IRMS coupled with an Isotope Ratio Mass Spectrometry (DeltaV A) at the Universidad de la Coruña. C_org_ isotopic signature of potential sources of C_org_, seagrass (*Zostera noltii*) and saltmarsh (*Halimione portulacoides*), were measured in biomass samples (2 replicate per species) collected from the same estuary and an estuary nearby, respectively.

Sediment accumulation rates were obtained from concentration profiles of ^210^Pb, determined by alpha spectrometry through the measurement of its granddaughter ^210^Po, assuming radioactive equilibrium between both radionuclides. About 100–200 mg aliquots of each sample were spiked with ^209^Po and microwave digested with a mixture of concentrated HNO_3_ and HF. Boric acid was then added to complex fluorides. The resulting solutions were evaporated and diluted to 100 mL 1 M HCl and Po isotopes were auto plated onto pure silver disks. Polonium emissions were measured by alpha spectrometry using PIPS detectors (CANBERRA, Mod.PD-450.18 A.M). Reagent blanks were comparable to the detector backgrounds. Analyses of replicate samples and reference materials were carried out systematically to ensure the accuracy and the precision of the results. The supported ^210^Pb was estimated as the average ^210^Pb concentration of the deepest layers once ^210^Pb reached constant values. Then, excess ^210^Pb (^210^Pb_xs_) concentrations were obtained by subtracting the supported ^210^Pb from the total ^210^Pb. Age model of the sediment depth profile record was obtained by modeling the ^210^Pb_xs_ concentration profiles along the accumulated mass at each site. The model age of the sediment record was estimated using the Constant Flux: Constant Sedimentation model (CF:CS^60^). In order to assess the impact of the bridge construction on the biogeochemical properties of the saltmarsh soil, we compare all biogeochemical properties across two sections of the cores, divided based on the results of ^210^Pb dating: sediments accumulated before and after the building of the bridge (i.e., before *vs.* after 1978).

## Data Availability

The datasets generated during and/or analyzed during the current study are available in the public repository DIGITAL CSIC (DOI: https://digital.csic.es/handle/10261/273128).
